# Draft Genome Sequences of the Type Strains of Six *Macrococcus* Species

**DOI:** 10.1128/MRA.00344-19

**Published:** 2019-05-09

**Authors:** Shahneela Mazhar, Eric Altermann, Colin Hill, Olivia McAuliffe

**Affiliations:** a Teagasc Food Research Centre, Moorepark, Fermoy, Cork, Ireland; b School of Microbiology, University College Cork, Cork, Ireland; c AgResearch, Animal Science Group, Palmerston North, New Zealand; d APC Microbiome Institute, Cork, Ireland; e Riddet Institute, Massey University, Palmerston North, New Zealand; Broad Institute of MIT and Harvard University

## Abstract

We report here the draft genome sequences of Macrococcus bovicus ATCC 51825^T^, Macrococcus carouselicus ATCC 51828^T^, Macrococcus equipercicus ATCC 51831^T^, Macrococcus brunensis CCM4811^T^, Macrococcus hajekii CCM4809^T^, and Macrococcus lamae CCM4815^T^. The availability of the genome sequences of these species will enable cross-species comparison, which could lead to a more comprehensive understanding of organisms of the Macrococcus genus.

## ANNOUNCEMENT

The Gram-positive genus Macrococcus contains a total of 11 species, Macrococcus bovicus, Macrococcus carouselicus, Macrococcus equipercicus, Macrococcus brunensis, Macrococcus hajekii, Macrococcus lamae, Macrococcus goetzii, Macrococcus epidermidis, Macrococcus bohemicus, Macrococcus caseolyticus, and Macrococcus canis (1, 2). These species are disseminated in nature as animal commensals and are indicated to be the immediate antecedent of the Staphylococcus species (3). While staphylococci are widespread human pathogens, macrococci are defined to be avirulent (4). However, recent publications have indicated the possible pathogenic potential of *Macrococcus* strains isolated from human clinical samples. The draft genome sequences reported here are of the type strains of six *Macrococcus* species that were isolated from artiodactyl and perissodactyl hosts (5, 6).

Genomic DNA was isolated from overnight cultures grown at 37°C in tryptic soy broth (TSB; Becton, Dickinson and Company, Berkshire, England) using the UltraClean microbial DNA isolation kit (Mo Bio Laboratories, Cambridge, United Kingdom) per the included protocol. Genomic libraries were prepared with the Nextera XT DNA library preparation kit (Illumina, Inc., San Diego, CA, USA). The 2 × 250-bp paired-end read sequencing was performed on an Illumina HiSeq 2500 platform (MicrobesNG, University of Birmingham, UK). Reads were adapter trimmed using Trimmomatic 0.30 with a sliding window quality cutoff of Q15 (7). *De novo* assembly was performed on each sample using SPAdes version 3.7 using the program’s default parameters (8). The genome sequences were annotated with the NCBI Prokaryotic Genome Annotation Pipeline (PGAP) (9). *In silico* analysis of acquired antimicrobial resistance genes and virulence genes was conducted using ResFinder version 3.4, VirulenceFinder version 2.0, PathogenFinder version 1.1, and the Virulence Factor Database (VFDB) (10, 11). CheckM was used to calculate the completeness (all illustrated 99.6% completeness) and purity (range, 1.1 to 2.04% impurity) of the reads (12).

In all sequenced genomes, capsule (*cap*)-associated genes that are involved in phagocytosis evasion were identified. These genes involved in capsule biosynthesis illustrated DNA sequence identities in the range of 70 to 92% to those present in pathogenic strains of Staphylococcus aureus. Other putative virulence factors found were hemolysin III (*hly-III*), aureolysin (*aur*), and fibronectin-binding protein A (*fbpA*), among others. The sequencing and assembly statistics of the draft genome sequences are shown in Table 1. The genome sequences of these species could facilitate a better understanding of the biology of these organisms and a comprehensive understanding of the genus *Macrococcus*, which in turn could contribute to a greater understanding of antibiotic resistance acquisition and the pathogenic potential of the genus Staphylococcus.

**TABLE 1:** Genome characteristics of the six type strains of the *Macrococcus* genus

Strain	GenBank accession no.	SRA accession no.	Draft genome size (bp)	G+C content (%)	No. of contigs	Coverage (×)	*N*_50_ (bp)	No. of CDS^*a*^
*M. bovicus* ATCC 51825^T^	SCWF00000000	SRR8448136	2,087,234	44.25	46	217	146,194	2,191
*M. hajekii* CCM4809^T^	SCWE00000000	SRR8448137	2,052,566	40.05	61	88	1,051,403	2,195
*M. carouselicus* ATCC 51828^T^	SCWD00000000	SRR8448134	2,069,165	43.83	34	83	1,168,712	2,153
*M. equipercicus* ATCC 51831^T^	SCWC00000000	SRR8448135	2,154,579	43.58	205	131	1,168,712	2,326
*M. lamae* CCM4815^T^	SCWB00000000	SRR8448132	2,031,524	40.28	73	156	102,971	2,095
*M. brunensis* CCM4811^T^	SCWA00000000	SRR8448133	2,089,391	41.59	83	122	99,997	2,184

a CDS, coding sequences.

### Data availability.

Genome sequence data of the six *Macrococcus* species were deposited into NCBI GenBank and the Sequence Read Archive (SRA) under BioProject number PRJNA515496. The accession numbers are listed in Table 1.
